# Preventive measures for fire-related injuries and their risk factors in residential buildings: a systematic review

**DOI:** 10.5249/jivr.v11i1.1057

**Published:** 2019-01

**Authors:** Mohammadreza Shokouhi, Khadijeh Nasiriani, Zahra Cheraghi, Ali Ardalan, Hamidreza Khankeh, Hosein Fallahzadeh, Davoud Khorasani-Zavareh

**Affiliations:** ^*a*^Department of Emergency & Disasters Health, Faculty of Health, Shahid Sadoughi University of Medical Sciences, Yazd, Iran.; ^*b*^Deputy of Treatment, Hamadan University of Medical Sciences, Hamadan, Iran.; ^*c*^School of Nursing and Midwifery, Shahid Sadoughi University of Medical Sciences, Yazd, Iran.; ^*d*^Department of Epidemiology and Biostatistics, School of Public Health, Hamadan University of Medical Sciences, Hamadan, Iran.; ^*e*^Department of Health in Disaster and Emergencies, School of Health, Tehran University of Medical Sciences, Tehran, Iran.; ^*f*^Research Center in Emergency and Health, University of Social Welfare and Rehabilitation Sciences, Tehran, Iran.; ^*g*^Department of Biostatics and Epidemiology, Research Center of Prevention and Epidemiology of Non-Communicable Disease, Faculty of Health, Shahid Sadoughi University of Medical Sciences, Yazd, Iran.; ^*h*^Safety Promotion and Injury Prevention Research Center, Shahid Beheshti University of Medical Sciences, Tehran, Iran.; ^*i*^Department of Health in Disaster and Emergencies, School of Public Health and Safety, Shahid Beheshti University of Medical Scienc-es, Tehran, Iran.

**Keywords:** Fires, Residential buildings, Preventive measures, Injury

## Abstract

**Background:**

Every year, a large number of people lose their lives or become injured seriously as a result of fires. Fires in buildings pose a great threat to resident safety. The aim of this systematic review is to identify preventive measures for fire-related injuries in residential buildings, taking into account associated risk factors.

**Methods:**

In this study, a systematic review was performed of all studies conducted in the field of residential building fires, influencing factors and available safety procedures. From the earliest record up to 7 July 2017, databases of PubMed, Web of Science/Knowledge, and Scopus were searched and selected articles included in the study.

**Results:**

A total of 5,613 published articles were examined, of which 30 were finally found to meet the inclusion criteria. The findings of the study were included in two main groups of preventive measures and risk factors for residential building fires and related injuries. Regarding preventive measures, the factors to reduce the risk of fire-related injuries raised in the studies under review included rule amendments, changes and modification of the environment, behavior change such as emergency evacuation during fire occurrence, improvements to emergency medical services, and awareness-raising. Also, many of the studies showed that areas with a large number of young children, older people, people with physical and mental disabilities, alcohol and drug addicts, smokers, single-family households and low-income families were particularly at risk of fire-related injuries and deaths.

**Conclusions:**

There are features in residential buildings and attributes among residents that can be related to fire hazard and fire-related injuries and deaths. The most important point of this study is to focus on preventive strategies including environmental modification, promotion of safety rules and changes in risk behavior among residents. Policy makers should pay more attention to these important issues in order to promote safety and injury prevention in relation to building fires.

## Introduction

Fire can have critical consequences for human society due to the damage it causes to buildings and infrastructure. Also, a large number of people lose their lives or become seriously injured as a result of fires.^1^ According to World Health Organization (WHO) statistics, more than 300,000 deaths are caused annually by fire-induced burns and more than 95 percent of these deaths occur in low- and middle-income countries (LMICs). ^2^ Building fires are believed to be a major threat to the safety of building occupants ^3^ and are mostly caused by people’s behavior. ^4^ In large residential complexes with numerous residents, in particular, fires can lead to increased injury or death due to emergency evacuation difficulties. This issue has been confirmed in various studies. ^5-7^ Therefore, the proper and safe design of buildings to protect human life in the event of fire is of great im-portance. ^8^ Studies indicate that large fires have occurred in build-ings with no or substandard fire alarm systems. Surveys conducted also indicate that at least 75% of fires are preventable. ^9^

According to the United States Fire Department, there is a fire in a residential area every 85 seconds and these fires account for almost 80 percent of all fire-related deaths.^6,7^ In London, 78% of deaths from unintentional fires are related to fires in residential areas.^10^ Fire-related injuries are one of the major causes of death and disability.^7^ In recent decades, even in high-income countries (HICs), despite the reduction in mortality rates, fire-caused deaths are still regarded as a major concern. ^11^ Between 2007 and 2010, approximately 39% of fires in China occurred in residential areas. Thus, considering the high possibility of death and injury and the financial losses incurred as a result of fires in residential buildings, more attention should be paid to this issue. ^1^

Previous studies have shown that most fire events are mainly caused by unsafe human behavior. In this regard, a definite way of reducing injuries is to control the unsafe behavior of individuals, This can be achieved by promoting safety activities.^12^ Indeed, promotion of practices related to fire prevention, rescue and evacuation training and revision of laws in recent years has already helped to reduce the number of fires in residential areas and related mortality. ^13^

Given, on the one hand, the importance of fires and safety considerations to prevent fires as well as to protect building residents and mental and physical well-being and respect residents’ status, and, on the other hand, the lack of systematic review studies that consider preventive measures and the risk factors to be considered, a systematic study of the influencing factors and safety procedures associated with fire injuries in residential buildings can be very helpful. It can increase knowledge of fire prevention and control actions taken in the world and help formulate guidelines and preventive regulations to combat fires in residential buildings and related injuries and deaths. The aim of this study is to identify preventive measures to combat injuries from fires in residential buildings, taking into account the associated risk factors.

## Methods 

**Search strategy**

In this study, in order to extract the required data from the databases, articles published in three prestigious Latin databases were used: Web of Science/Knowledge, PubMed and Scopus. All databases were searched in English from the earliest record up to 7 July 2017. For this purpose, in the search strategy, a combination of the following keywords was used: ("Dwellings fires" OR "Dwellings fire" OR "house fires" OR "house fire" OR "residential fires" OR "residential fire" OR "home fires" OR "home fire" OR "household fires" OR "household fire" OR "building fires" OR "building fire") AND ("Asphyxia" OR "injuries" OR "death" OR "mortality" OR morbidity OR "fatal" OR "burn" OR "damage").

The articles used in this study were selected in three stages. In the first stage, having searched for relevant articles in the databases, two researchers selected articles independently of one another. Ultimately, agreement was reached on issues that were controversial, based on scientific discussion. The expected agreement value (Kappa statistics) was 82.5. In the first stage, based on relevancy, citation information, together with a summary of all the articles extracted from the databases, was transferred to management software references (EndNote). After removing duplicated studies, the remainder were reviewed based on the title and summary of the study for inclusion in the review. The titles of selected articles were reviewed, and articles that were irrelevant to the main research topic were omitted. In the second stage, the remaining paper abstracts were studied, and related studies to the main aim of project were selected. In the third stage, the full texts of papers were studied and then all the papers which focused on the factors influencing injuries caused by fires in residential buildings as well as safety measures were selected. Then, after being assured of the relevance of the selected articles, studies that met the inclusion criteria were selected and then evaluated and the data were extracted. In order to obtain more material, the list of references in the papers was reviewed and related articles were added to the study. If the paper abstract was not available, or if it was impossible to determine whether the paper could be chosen on the basis of the abstract alone or not, the total paper was reviewed. After determining the final articles, the required information, such as title, study design, location and type of data, was studied. Finally, the risk factors and preventive measures were extracted from the text of the selected articles, and were entered into Excel 2010 to be compared.

**Inclusion and exclusion criteria**

All of the descriptive, observational and intervention studies related to building fires and injuries incurred were investigated regardless of the time of the study, the place and language of publication. The criteria of the selection of the articles included studies focusing on the influencing factors and safety measures related to injuries from fires in residential buildings. One key selection condition of these articles was that the fires had occurred in residential buildings. Non-residential building studies were excluded. Also, articles that did not mention the causes of injury and death or preventive measures were excluded from the study. Articles with abstracts in English were also included in our study. In terms of time, all studies that were indexed from the beginning of the above mentioned databases were included in the study.

**The type of population surveyed**

All people who live in residential buildings and were at risk of potential fires were studied.

**Data collection and data analysis**

**How to investigate – quality of selected studies (or risk of bias):**

In this study, the STROBE checklist was used to evaluate the quality of the reporting of included studies in this review.^14^ The criteria that were used included: the inclusion and exclusion criteria; the time and place of study; how to measure the outcome(s); how to measure the exposure, and the study design.

**Selection of studies and extracting data**

In order to ensure the correct selection of relevant articles in accordance with the inclusion criteria, three researchers (MRSH, DZKH and ZCH) independently selected articles. The name of the authors, journal name and the results were not hidden for these researchers. In cases where the researchers had conflict, the final decision was reached through negotiation. After selection, required variables, including study design, time and place of study, and most important result, were entered in a check list (Table 1).

**Table 1:** Design, study type, country and year of publication of articles entered in the systematic study on preventive measures and their risk factors for Residential-Building-Fires related Injuries (RBFIs) from the earliest record indexed up to 7 July 2017.

Design	Country	year	Most important results
**Cross sectional**	Sweden	2017	Smoking and alcohol consumption; fires that started in the bedroom or living room, and fires started in beds/sofas/armchairs and clothing are most important risk factors & cooperation between the various municipal departments; automatic stove turn-off devices, and enhanced knowledge of fire-related death are the most important pre-ventive measures for RBFIs.
**Cross sectional or Case control**	US	2017	Smoke alarms are most important preventive measures for RBFIs.
**Cross sectional**	US	1987	Inflammable material, smoking in bed are most important risk factors & increase the number of functioning smoke detectors, promoting safe storage of flammable liquid are the most important preventive measures for RBFIs.
**Cross sectional**	US	1998	Physical disability is most important risk factor & smoke alarm, reduction of residential fire hazards, the design and practice of fire escape plans and fire safety education are the most important preventive measures for RBFIs.
**Cross sectional**	US	2011	Fires started by smoking and lack of smoke alarms are most important risk factors & operating smoke alarms, wet pipe sprinklers, and fire safety education are most im-portant preventive measure for RBFIs.
**Cross sectional**	US	2007	Occupants with alcohol problems are most important risk factor & presence of a fire extinguisher is most important preventive measures for RBFIs.
**Cross sectional**	US	1992	Cigarettes are the most important risk factors for RBFIs.
**Cross sectional**	US	2005	Cigarettes are the most important risk factors for RBFIs.
**Cross sectional**	Canada	1993	Longer fire department response time; smokers’ material; children playing with matches/lighters & alcohol consumption are most important risk factors for RBFIs
**Cross sectional**	Taiwan	2008	Discarded cigarette, smoke in the bedrooms are most important risk factors & plan escape; improve fire-use behaviors; installation of individual fire alarm system & fire safety education are most important preventive measures for RBFIs.
**Interventional study**	US	2010	Smoke alarms; fire safety education; escape planning; broad health education are the most important preventive measures for RBFIs.
**Qualitative**	UK	2000	Discharge of patients without follow-up; discharged patients before treatment are the most important risk factors & self-extinguishing cigarettes; non-flammable furnishings and building materials & use of sprinkler systems are most important preventive measures for RBFIs.
**Cross sectional**	UK	1998	Reduced fabric flammability; improvements in emergency services and medical care; smoke alarm installation are the most important preventive measures for RBFIs.
**Cross sectional**	US	1989	Impact of hearing and visual impairment; Impaired tissue regeneration; impaired sense of smell; arthritic hands, and weak grip are most important risk factors & promotion of the use of smoke detectors and automatic sprinklers are the most im-portant preventive measures for RBFIs.
**Cross sectional**	Netherlands	2009	More frequent evacuation drills are the most important preventive measures for RBFIs.
**Cross sectional**	Australia	2013	Intoxication; households with smokers are most important risk factors & smoke alarm ownership is most important preventive measure for RBFIs.
**Cross sectional**	US	2006	Fire origin in kitchen and bedroom & misuse of heaters by children are most important risk factors for RBFIs.
**Cross sectional**	US	2001	Houses without functioning smoke detectors; fires that began in bedrooms, and fires ignited by electrical wiring are most important risk factors & distribution of smoke detector is most important preventive measure for RBFIs.
**Cross sectional**	Scotland	1999	Alcohol and smoking materials are most important risk factors functional smoke detectors; escape from the fire; water to extinguish the fire; use of safer centrally heated systems, and stop smoking in bed are most important preventive measures for RBFIs.
**Case–control study**	Denmark	1998	Somatic and psychiatric conditions, and disabled people, intoxication by alcohol are most important risk factors & self-extinguishing cigarettes and installing sprinklers are most important preventive measures for RBFIs.
**Cross sectional**	Denmark	1998	Alcohol intoxication; disability; smoking in bed are most important risk factors & self-extinguishing cigarettes; use of fireproof materials in furniture and clothing and smoke-alarms are most important preventive measures for RBFIs.
**Cross sectional**	New Zealand	2005	Disabilities are most important risk factor & smoke alarms; egress standards, and building code reform are most important preventive measures for RBFIs.
**Cross sectional**	Indian	1994	Smoking in bed and use of a wood stove are most important risk factors & installation of smoke detectors and automatic sprinkler system are most important preventive measures for RBFIs.
**Case control**	US	1993	Absence of a smoke detector and use of alcohol or other drugs are most important risk factors & increasing the number of exits; use of sprinklers and smoke detectors are most im-portant preventive measures for RBFIs.
**Cross sectional**	Scotland	1995	Flammable liquids such as petrol; children left alone and alcohol intoxication are most important risk factors & escape from fire and smoke alarm legislation are most important preventive measures for RBFIs.
**Cross sectional**	Scotland	1997	Alcohol intoxication is most important risk factors & fire safety education and prevention campaigns are most important preventive measures for RBFIs.
**Cross sectional**	Scotland	1996	Children playing with matches, etc.; alcohol intoxication; unable to escape; careless disposal of smoking materials, and access to matches are most important risk factors & preventing the onset of the fire; early detection coupled with escape in the event of fire and prompt emergency medical treatment of casualties are most important pre-ventive measures for RBFIs.
**Case control**	Australia	2015	Failure of electrical appliances and discarded cigarette materials are the most im-portant risk factors & an activated smoke alarm and a clean and tidy home are the most important preven-tive measures for RBFIs.
**Cross sectional**	Australia	2017	Discarded cigarette and failure of electrical appliances are most important risk fac-tors & smoke alarm installation is the most important preventive measure for RBFIs.
**Cross sectional**	US	2014	Decreased cigarette consumption; creation of flammability standards for mattresses and upholstered furniture; increase in smoke detectors and sprin-kler systems; improvements in trauma systems and burns and critical care are the most important preventive measures for RBFIs.

## Results

**Figure 1:**
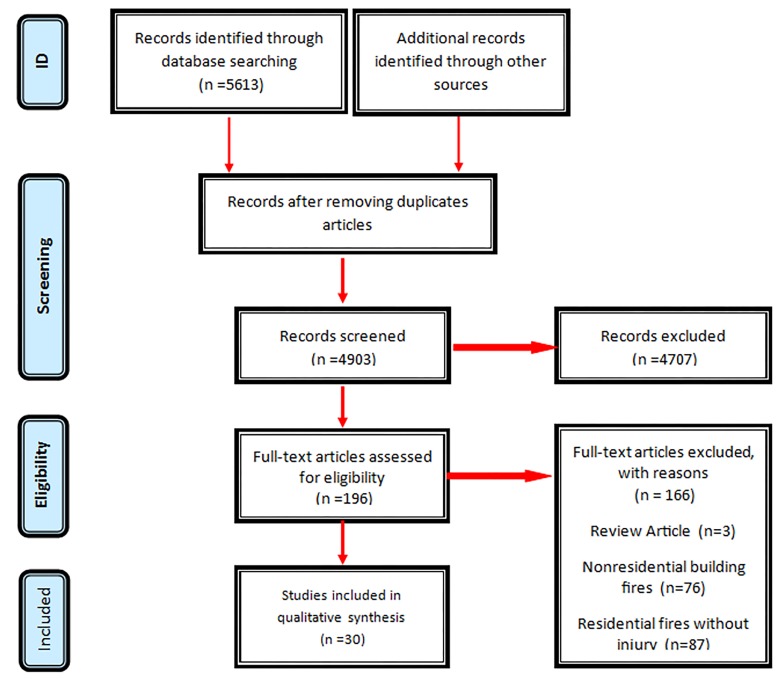
Selecting articles according to the risk factors and preventive measures of building fire injuries from the first record up to 7 July 2017 by PRISMA 2009.

In the initial search and using online search strategy, a total of 5,613 studies were identified that, together with articles taken from other sources, reached a total of 5,659 studies. After retrieving all the articles and removing 756 duplicate studies, a total of 4,903 studies were found. Among them, 196 articles remained that were based on the study aim, which were included after reviewing the titles and abstracts as well as application of inclusion and exclusion criteria. After reviewing the full text of articles, 30 studies were based on all criteria and ultimately these studies were selected for final analysis. The process of retrieving and selecting articles is shown in Figure 1.

**Figure 2:**
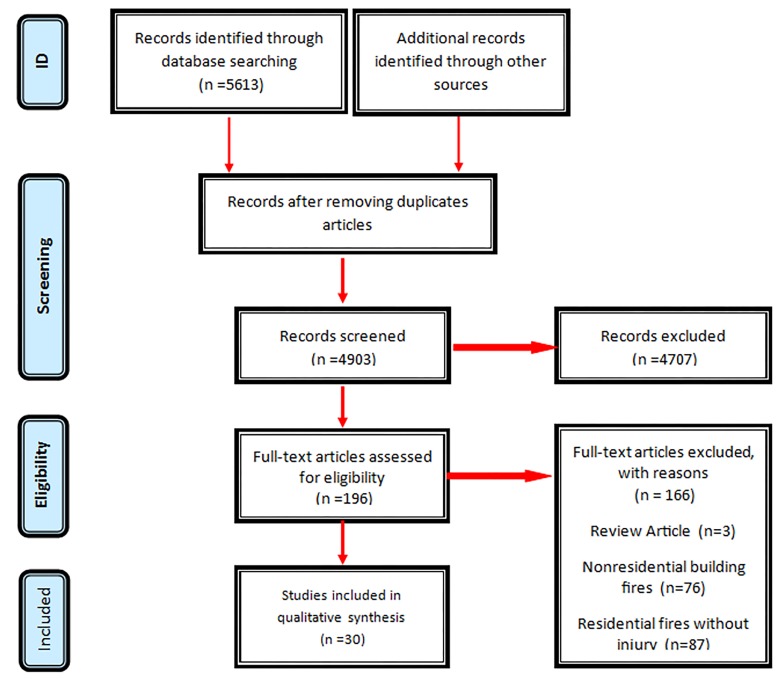
Quality Assessment of studies by STROBE checklist based on the systematic review of preventive measures and risk factors for residential building fires and injuries from first ever record up to 7 July 2017.

As mentioned in the methodology, regarding the STORBE checklist, quality assessment was conducted. Based on our assessment, the studies were divided into three groups: low risk of bias (to consider 100% of STROBE checklist), moderate risk of bias (to consider up to 85% of STROBE checklist) and high risk of bias (to consider less than 70% of STROBE checklist). Results of the risk bias investigation are detailed in Figure 2.

Of 30 papers having the requirements to be included in the study, four were case studies, 25 cross-sectional and one study was interventional. Also, 12 studies were carried out in the United States, 4 studies in Scotland and 3 studies in Australia, and others in the UK, India, Taiwan, Denmark, Sweden, New Zealand and the Netherlands. The current study has reviewed 1) risk factors and injuries related to building fires and 2) fire-related preventive measures in residential buildings.

**Risk factors and injuries related to building fires**

In the selected 30 studies, risk factors for fires and injuries in residential areas were investigated. Risk factors were classified in three categories related to residents, building and fires (Table 2).

**Table 2:** Key distinguishing risk factors for residential building, injuries and deaths.

Category	Subcategory
**Risk factors associated with individual characteristics**	Disability, physical and mental illnesses
	Drug and alcohol addiction
	Households with a higher number of residents under the age of 5 years and aged 65+ years
	Households with high vulnerability residents
	Households with non-working residents
	Living alone
	Low awareness
	Unemployment
**Risk factors related to residents' behavior**	Impairment by alcohol/drugs
	Smoking
	Carelessly discarded cigarette butts are a frequent cause of fires
	Children playing with fire
	Leaving children at home alone
	Approaching the fire
	Use of inappropriate clothing during cooking
	Negligence and neglect
**Risk factors associated with building**	Older buildings
	Cluttered buildings and buildings in a state of disrepair
	High-rise building
	Building without fire detection & fire extinguishing systems
	Building with gas piping
	Building with unsafe electrical system
**Risk factors related to social-economic conditions**	Unavailability of fire service and time-consuming
	Inadequate safety education in the community
	Poverty and low income of households
	Minorities
	Cultural poverty
	Overcrowded households
	Social inequality
	Insufficient supervision
	Families with smokers and alcoholics
**Risk factors related to fire circumstances**	Fires ignited in the living room
	Fires ignited in the bedroom
	Fires ignited in the kitchen
	Fires ignited from the bed and furniture
	Fires ignited at night
	Fires ignited during winter months
	Fires ignited in the room where the person is sleeping/alone
	Fires ignited from heating and electrical appliances
	Fires ignited from smoking materials/cigarettes
	A huge fire without the opportunity to leave

**Risk factors associated with residents**

Risk factors associated with humans in the event of fires and injuries related to two aspects: characteristics of residents and residents' risky behavior.

**Characteristics of residents**

Many characteristics of residents were evaluated in this study including age,^2,9,13,15-29^ race and ethnicity^9,13,16^ mental health,^21,24,30,31^ physical illness,^21,24,27,30^ drug intake,^22,24,27,31^ job status,^30,24^ and marital status.^30,32^ Among the risk factors related to characteristics of residents, age was allocated the highest risk factor as emphasized in most studies. Older people over the age of 65 years,^2,9,13,16-19,21,22,27,33,34^ and children less than 5 years old,^2,15,23,33^ were mentioned as the groups at greater risk. Diseases and physical disabilities were accounted for as risk factors in several studies.^21,24,27,28,30^ Also, among these factors, physical and cognitive impairments following alcohol use^20,16-18,22,24,25,28,30,31^ and drug abuse^22,24,27,31^were mentioned in most studies. 

**Risky behavior of residents**

Smoking^9,17,18,20,22,25,27^ especially in bed,^17,20,25,27^ alcohol consumption,^9,16-18,20,22,24-27,31,32^ inappropriate use of and playing with fire by children,^9,26,33,35^ and dropping cigarette butts on the floor,^15,17,24,26,31^ were indicated in several studies. Two studies showed that households with more smokers are at greater risk of fires.^16,32^ Seven other studies examined the association between smoking and fires-related injuries and deaths.^9,17,18,20,22,25,27^ Several studies have focused on the link between simultaneous smoking and alcohol consumption with increased injuries and deaths caused by fires.^16-18,25,30^

**Building-associated risk factors**

**Structures-related features**

Several studies^9,21,22,24,27^ have pointed out the relationship between building features and increased risk of fire-related injuries. The most important of which were high-rise buildings^22^ and buildings with substandard construction materials.^24,27^ A study shows that buildings with less than two exits are more at risk of death from fires.^21^

**Equipment and setting features**

Several studies have identified the relationship between the non-installation of smoke detectors with risk of injury and death.^9,19-22,24,33^ Many studies have examined the relationship between the presence of flammable materials and fires and increased risk of injury and death. ^21,23,31^

**Socio-economic factors**

Socio-economic status, including low income, was examined in several studies.^2,9,18^ In these studies, low income was related to an increased risk of injury and death caused by fires. The unavailability of the fire service^33,27^ and safety training^18,29^ are also risk factors taken into consideration. Bad socio-economic status,^2,16,18,23,26,34^ rural agricultural areas,^36^ households with smokers and alcoholics^9,17,18,20,22,24-26,28,30-35^ were also considered. The relationship between employment status and the risk of house fires in two studies was investigated.^2,24,30^ Both these studies showed that families with unemployed members were more vulnerable to fires. Failure to properly monitor injuries from fires in the community was examined in one study and was found to increase the likelihood and severity of ensuing injuries.^29^

**Fire-related circumstances**

Many studies have examined the relationship between the kind of fires and the risk of injury and death. These studies show that if a fire starts in the bedroom^18,26^ or living room^9,18^ or kitchen^26^there is a greater risk of injury and death. Several studies show that fires caused by smoking and thermal materials^9,17,26,33,35^ can cause more injuries.

**Fire-related preventive measures in residential buildings**

In the 30 selected papers, preventive measures against fires and injuries and mortality were studied in residential areas. The measures were classified into five categories: rule reform, environmental modification, the actions and behavior of residents, rescue and medical care system promotion and education (Table 3).

**Table 3:** Preventive measures related to building fires and injuries based on the systematic review of preventive measures and risk factors for residential building fires and injuries from first ever record up to 7 July 2017.

Category	Subcategory
**Rule modification**	Enforcing cigarette safety regulations
	Creating rules of installing alert system
	Planning for the use of local people
	Applying the decision-making system for relief and rescue
	Financial incentives to install the safety system
	More coordination between urban departments
	Modification of construction and engineering rules
	The prosecution of mothers who leave their children alone
**Environment modification (structure and content of building)**	Use of fire extinguishing and alert system in buildingsSupplies of standard and non-flammable building materials, furniture and interior decoration,
	Safe emergency exit design
	Upgrading of electrical equipment
	Use of safe ovens (auto shut-off)
	Installing a safe heating system
	Improving the quality of fire control systems
	Safe storage of fluids and flammable materials
	Gridded window upgrade
	Creating enough light in the building
	Cleaning and tidying inside buildings
**Residents’ safe behaviors and actions (safety management)**	Prevention of building fires and reducing their risks
	Planning for emergency exit and safe location
	Promote safe fire-related behavior
	Periodic checking of heating and power supplies and safety systems
	Performing emergency evacuation maneuvers
	Quitting smoking, especially in bed
	Escape when fire breaks out
	Not wearing loose clothes while cooking
	Reducing alcohol consumption
	Use of fireproof clothes
	Safety recommendations for children
	Use of smoke protection equipment
	Use of safe cigarettes
	Closing off the staircase as well as doors and windows during sleep to prevent smoke emissions
**Upgrading rescue and relief system and medical care**	Treatment system promotion (hospital and pre-hospital)
	Rapid implementation of firefighting services
	Trauma system promotion
	Trauma system promotion
	Monitoring and treating psychiatric patients
	Reducing response time
**Education**	Fire safety training (how to use fire safely, how to use fire extinguishers, how to use fire escape facilities
	Promotion of fire prevention program
	Creating training and safety campaigns and general information
	Creating cigarette and alcohol quitting campaigns
	Practical drills for dealing with fire
	Strengthening relief and rescue training
	Increasing knowledge about fire-related deaths
	Teaching children about the dangers of matches and fire

**Actions related to legislative reform**

Many characteristics associated with law reform and law enforcement regarding the prevention of fires and injuries were evaluated in this study. These include laws related to the installation of warning systems and safety equipment,^2,15^ the implementation of safety rules related to smoking,^9,13^ laws related to planning and coordination between municipal departments^18^ and also amending construction and engineering laws ^28^ are the most important preventive measures with respect to legislative reform.

**Environmental modification measures**

Among the preventive measures related to environmental modification and improvement, the following were the most important findings: the use of fire detection and fire extinguishing systems, such as installation of automatic sprinklers,^9,13,19-21,27-30,35^ active smoke alarms,^2,9,13,16,17,19-24,27,28,30,31,36-39^ and other fire extinguishing systems in buildings, ^17,32^ design of emergency exits,^15,17,21,28^ installation of central heating systems.^35^ Among these measures, the use of alarm systems and fire extinguishers was investigated in 23 studies, and indeed all except seven studies referred to the necessity of using these systems. Automatic sprinkler system construction and installation as well as the use of active smoke-warning systems were noted in most cases. In two studies, the roles of fire alarm systems in buildings were noted.

**Measures related to residents’ behaviors**

Many studies looked at the behavior of residents in connection with escape planning and emergency exits and going to a safe place during fires.^2,17,37,40^ Also, not smoking, especially in bed,^35^ reduction of alcohol consumption,^40^ use of protective equipment against smoke,^35^ closing the doors and windows while sleeping to prevent the spread of smoke^17^ as characteristics associated with the behavior of building occupants were examined in numerous studies.

** Rescue system and medical care measures**

A total of seven studies examined the relationship between the promotion of rescue systems as well as medical care in reducing the risk of injury and death. In these studies, improving burns care,^13^ upgrading the emergency treatment of victims,^20,23^ doing a quick fire aid service and primary medical treatment,^30^ were highlighted. Moreover, improvement of trauma systems and fire victim care were examined in six studies^13,17,20,23,30,39^ and the importance of their role in reducing injuries and deaths after a fire was emphasized. In addition, the importance of improving the quality of firefighting and rescue services in injury reduction was highlighted in two studies.^17,30^

**Training-related activities**

Training in connection with fire safety (training in how to use a fire extinguisher and how to escape);^2,9,16,17,19,37^ conducting an emergency evacuation program,^2,38^ were mentioned in relation to fire safety training. Safety, training and public information campaigns and also creating campaigns for smoking and alcohol cessation,^30^ practical implementation dealing with fires,^30^ creating safety and prevention programs for target groups,^27^ reinforcement of rescue training,^17^ school education to educate children about the dangers in society,^35^ were mentioned as preventive measures related to education in the studies.

## Discussion

This systematic review identified the results of residential building fires and its related mortality, in addition to risk factors, greater focus on the prevention of fires and injuries caused by it, taken from the best scientific evidence. Based on these factors, evidence-based preventive measures regarding building fires and injuries can be designed in the future. The most important risk factors in most studies covered the following: high-risk behaviors of residents during fires; physical and mental disabilities and the disabilities associated with age; non-standard structural and non-structural factors in residential buildings; factors related to failure of fire alarm system installation; factors associated with unsafe equipment and furniture setting; the economic problems of the community and residents of residential buildings; social and cultural factors; and low perceived risk of the starting and spreading of fire. Also, prevention measures against fires and injuries in residential buildings that were extracted from the studies include: laws reform; modifying the environment; safety measures and human behavior; promotion of aid systems and promotion of safety education in the community and awareness raising. According to the results obtained, the elderly and children, especially children under five, compared to other age groups, are more vulnerable to fires due perhaps to difficulty in physical movement and a lack of appropriate response to fire. It seems these age groups should be paid more attention to during the construction of infrastructure. Evidence also shows that the global population of the elderly will be more in the coming years.^41^ Therefore, it is necessary for this group to be looked at in terms of safety against fires. Physical and mental illnesses and addiction to alcohol and drugs are associated with the increased risk of fires, including related injuries and deaths. Thus, these groups must be taken into account and make more use of adequate safety equipment in their buildings.^21^

Risks are far higher in smoking households than in no-smoking households^16,32^ and this can be due especially to smoking cigarettes around the bed.^17,20,25,27^ Therefore, avoiding smoking, especially while in bed as well as fire safety cigarettes can reduce the likelihood of such incidents and injuries. In many European countries smoking is forbidden in enclosed spaces, and people are forced to smoke outside their homes. Using this experience, especially in LMICs, the incidence of fires occurring after indoor smoking can be prevented.^42^ Law reform and education to change people's behavior on the issue of smoking and not throwing cigarette butts around the home play an important role in promoting safety, reducing fires and associated injuries.

Several studies show that the risk of fires and associated morbidity and mortality among alcoholics is much greater due to the inability of people to react appropriately during fires.^16-18,20,22,24-28,31,32^ Moreover, social and low economic variables may play a role in damaging this group and affect other factors; for example, alcoholics living in buildings that do not have the necessary safety against fires.^43^To prevent injury attributable to alcohol, we need educational campaign to change people's behaviors and prohibit the use of alcohol.

Lack of safety systems such heat and smoke detectors, and fire extinguishing systems increases the possibility of injury and death from fires in buildings. A system approach is needed to be considered in such incident, as it already pronounced to other type of injuries.^44^ By modifying the legislation regarding the installation of these systems, fire-related casualties can be reduced.^2,13,17,19-25,27-31,35-39^ Properly functioning smoke detectors control and reduce the spread of fires and thus create protection against them. Although detector installation is applied in most HICs as a principle, the use of detectors in LMICs is not widespread. It seems that these countries should also develop a requirement for smoke detector installation in their building codes for both buildings under construction and existing buildings.

Inappropriate setting of interior equipment and use of unsafe equipment can increase the risk of fires in homes and it seems that the use of equipment such as gas containers, primus and oil and gas heaters in LMICs must be banned, as it is as HICs, which have stopped the use of these devices over the past years. Use of safe equipment and the elimination of hazardous and unsafe equipment play an important role in the prevention of injuries caused by fires.

The relationship between low socio–economic status, such as low income, and an increased risk of injuries and deaths caused by fires was reviewed in several studies.^2,18^ The link can be due to failure to prioritize safety as well as a lack of financial resources to buy safety equipment such as smoke detectors. This risk is much higher in LMICs than in HICs, because of economic and political problems in LMICs.^45^ Also, in LMICs, there are low restrictions on alcohol consumption.^45,46^ With regard to much of the fire- related injury in LMICs, preventive measures such as the distribution of safety equipment to households most at risk can be used.^46^ Another cause of increased risk of injury from fires in LMICs is the unavailability of appropriate firefighting services^27,33^ and the lack of adequate safety training.^18,29^ Given that the weakness in the rescue and medical care also increases the likelihood of injury, upgrading fire rescue and first aid practices and medical care can be appropriate preventive measures in this regard. Lack of safety training in the community is one risk factor associated with fires and it is better to resolve the issue through fire safety training such as learning how to use an extinguisher^2,16,17,19,37^ and emergency evacuation maneuver ^2,38^ for the target groups. The present study highlights the role of promoting safety education and raising awareness on the prevention of fires and injuries. Then, we can predict residents’ behavior during fires based on the capability and knowledge of building residents.^47^

The roles of emergency exits and escape during fires in reducing injury and death were taken into account in most studies.^17,23,35,37,39^ But in many LMICs, the importance of emergency exits has not been considered even in new constructions and high-rise building. In order to overcome this, it should be seriously considered as a problem even in most hospital.^48^ It suggests that more attention should be paid to saving the lives of building occupants in the event of fire. Although in many buildings that having already been constructed, it is not possible to install emergency exits; in recent years the use of various backpack emergency escape systems, which at the time of the fires can be used to rescue the occupants of a building, especially on the upper floors, has become common. ^49^ It has been long observed that people living on the upper floors tend to jump to escape the fire. So what seems to be important is that all buildings lacking emergency exits have to have alternative escape methods, similar to those mentioned above.

In the present study, the role of substandard equipment and construction materials has been highlighted in the start and spread of fires, as confirmed in another study.^11^ Fires can spread and can even result in the collapse of building. Concrete structures are more resistant to fire and collapse.^50^ The emphasis of the need for appropriate building infrastructure mentioned in the study is quite evident in the case of the Plasco building fire in Tehran, Iran. The building collapsed during the fire and rescue services for the victims proved fruitless. Therefore, considering the necessity and importance of planning for risk reduction against disasters and fires must be taken into account subjectively or objectively.^51^ The role of low-quality and substandard equipment in fires was emphasized in this study and the case of Grenfell Tower in London also shows that the fire was caused by a faulty refrigerator and spread quickly via highly flammable insulation material.^11^ Fires starting in the bedroom and during sleep cause increased injury as emphasized in another study and the increase of injuries can be due to fire igniting quickly on the bed and people not being able to evacuate before the fire spreads.^52^

It is noteworthy that most of the findings are drawn from HICs (28 studies), especially from the United States (12 studies) and there are fewer studies in LMICs. These studies show that homes without active smoke detectors, standard heating and electrical devices are constantly more vulnerable to fire-related mortality and morbidity. However, due to lack of sufficient study, it is not clear whether this type of household accommodation is at more risk of fire.

According to what has been mentioned previously, measuring the safety of buildings in terms of structural, non-structural and functional dimensions seems to be necessary to plan to meet building safety requirements. In a study, some of the indicators related to building safety were pointed out.^53^ A study conducted in Iran to evaluate the structural safety of hospitals during disasters highlighted the need for safety measures in buildings.^48^ These studies show that over the past three decades, significant improvement has been made in terms of safety (in the new regulations on the structural and non-structural section of building) and increased communication devices including mobile phones. Reduced smoking and increased use of detectors have also helped. Further investigation of a meta-analysis type on the effects of various factors on each other, the effectiveness of preventive interventions and programs of prevention programs, especially addressing the risk groups, including the elderly and children, is necessary to provide more evidence-based findings.

**Weaknesses and strengths of the study**

The major limitations of this study are unavailable full texts of some papers, outdated papers and non-English papers. However, in order to overcome it, the authors did try to find full text in various ways including communication with some other students in other universities abroad to provide them. Moreover, this study did try to define criteria that are used to present the data clearly in each step and the reader can evaluate the study easily.

One of the important strengths of this study is a comprehensive approach not only to risk factors but more importantly to preventive measures pertaining to mortality and morbidity with respect to fire-related consequences worldwide. Several studies on fires in buildings have been carried out around the world but the present study aims to show the risk factors associated with mortality and morbidity caused by fires in buildings, in addition to highlighting prevention measures. This study attempts to present risk factors and preventive measures based on the best scientific evidence to plan preventive measures; and to combat fires in buildings and associated injury and death.

## Conclusion

Fire-related incidents in residential buildings as well as their related mortality and morbidity are a major public health problem, but existing studies published in the field in recent years have been limited especially in LMICs; and most of the studies had an epidemiological approach.

This study showed that individual unsafe behaviors such as alcohol consumption and smoking, especially in bed as well as individual characteristics of residents such as disability, old and young age, along with risk factors associated with building structures, including the use of unsafe, flammable materials, as well as unsafe equipment and inappropriate placement of home appliances along with the lack of warning equipment are among the most important fire risk factors. Regarding the risk factors mentioned above, the results of this study show that, for the safety of building occupants, environmental modifications, such as the use of alarm and fire systems and the use of safe electrical and heating equipment in buildings and safe building materials along with legislative amendments including compulsory installation of alarm systems and preventing risky behaviors of the residents can all be effective.

The study points to the importance of designing and carrying out early interventions (to reduce the risk of fires in the family with the highest risk) and secondary interventions (improved viability in the event of fires for families which are at greater risk of injury and death). Such interventions are also useful for designers of residential buildings, rescue service officials and other individuals and organizations that play an important role in the prevention of fires.

**Acknowledgment**

The author would like to thanks firefighters, physicians and nurses involved in the provision of the data in this study.
